# NiO-PTA supported on ZIF-8 as a highly effective catalyst for hydrocracking of Jatropha oil

**DOI:** 10.1038/srep23667

**Published:** 2016-03-29

**Authors:** Jing Liu, Jing He, Luying Wang, Rong Li, Pan Chen, Xin Rao, Lihong Deng, Long Rong, Jiandu Lei

**Affiliations:** 1 Beijing Key Laboratory of Lignocellulosic Chemistry, College of Materials Science and Technology, Beijing Forestry University, Beijing 100083, P. R. China; 2 Key Laboratory for Biomechanics and Mechanobiology of Ministry of Education, School of Biological Science and Medical Engineering, Beihang University, Beijing 100191, P. R. China

## Abstract

Nickel oxide (NiO) and phosphotungstic acid (PTA) supported on a ZIF-8 (NiO-PTA/ZIF-8) catalyst was first synthesized and it showed high activity and good selectivity for the hydrocracking of Jatropha oil. The catalyst was characterized by SEM, SEM-EDS, TEM, N_2_ adsorption, FT-IR, XRD and XPS. Compared with the NiO-PTA/Al_2_O_3_ catalyst, the selectivity of C15-C18 hydrocarbon increased over 36%, and catalytic efficiency increased 10 times over the NiO-PTA/ZIF-8 catalyst. The prepared NiO-PTA/ZIF-8 catalyst was stable for a reaction time of 104 h and the kinetic behavior was also analyzed. This catalyst was found to bypass the presulfurization process, showing promise as an alternative to sulfided catalysts for green diesel production.

As global fossil fuel reserves diminish, there is a pressing need to develop renewable liquid fuels that are environmentally friendly^1^. Biofuels such as green diesel produced by hydroprocessing of plant oils typically display similar properties to petroleum diesel. They can be directly used in existing infrastructures with no modifications^2^. Various porous sulfided catalysts have been used to produce biofuels from plant oils^3^. NiMo^4^,^5^,^6^,^7^, CoMo^8^,^9^,^10^, Pt^11^,^12^ and Pd^13^,^14^ based catalysts with porous SiO_2_-Al_2_O_3_^15^,^16^ and zeolites^17^,^18^ support have been used for hydrotreating of plant oils^19^. These catalysts were successfuly used to convert renewable feedstocks into green diesel, stimulating exploration of the potential of other porous materials as catalysts. Due to their high specific area and porosity^20^,^21^,^22^,^23^, metal organic frameworks (MOFs) are appealing candidates for this type of conversion.

The selection of MOF supports significantly affects catalytic activity and product selectivity^24^,^25^,^26^,^27^. Zeolitic imidazolate frameworks (ZIFs)^28^,^29^,^30^, a subfamily of MOFs, is a microporous material with uniform small pores. A number of ZIFs exhibit permanent porosity, as well as a thermal and chemical stability^31^,^32^. The prototype system ZIF-8 has been proposed as a promising material in adsorption/separation^33^,^34^ and as a heterogeneous catalyst^35^. It has been reported that ZIF-8 showed excellent catalytic activity and selectivity for transesterification of DMC with DEC^36^, and catalyze transesterification of vegetable oil with significant activity^37^. ZIF-8 was also used as a catalyst for Friedel-Crafts acylation of anisole with benzoyl chloride and the reaction afforded a selectivity of 93–95% to the *p*-isomer^38^.

The design and preparation of several catalysts in ZIF-8 supported monometallic or bimetallic nanoparticles have been reported, since metal nanoparticles supported on ZIF-8 show improved catalytic activity and selectivity^39^,^40^. For example, Pd@ZIF-8 exhibitd excellent catalytic activity toward the catalytic reduction of *p*-nitrophenol to *p*-aminophenol in the presence of NaBH_4_ at room temperature^41^. Hydrogen reduction and CO oxidation of Au/ZIF-8 was performed to verify the structural stability of the framework under common preparation and application conditions used for supported metal catalysts^42^. The Fe_3_O_4_@ZIF-8 catalyst exhibited fair separation ability and reusability, which can be repeatedly applied for Knoevenagel condensations and Huisgen cycloadditions for at least ten successive cycles^43^. The NiRh supported on nitrogen-doped porous carbon (NPC) derived from ZIF-8 were synthesized and exhibited the highest catalytic activity and 100% hydrogen selectivity toward hydrogen generation from hydrazine^44^. The Pd/ZIF-8-derived carbon catalyst exerts extremely high catalytic activity and electrochemical stability for methanol electrooxidation^45^. Tuan T. Dang *et al.*^46^ reported PdNPs/ZIF-8 was a efficient heterogeneous catalyst for the aminocarbonylation of bromoarenes.

The high surface area and narrow pore distribution of ZIF-8 may lead to uniform distribution of highly dispersed nanoparticles, which may control catalytic activity and selectivity^47^. For example, when Pd, Pt, Ru, and Ir nanoparticles supported on ZIF-8 were applied to the hydrogenation reaction, they showed high activity and selectivity^48^. Guang Lu *et al.*^35^ reported that Pt, Fe_2_O_3_ and CdTe nanoparticles supported on ZIF-8 exhibited catalytic, magnetic and photoluminescence properties, respectively. Hailong Jiang *et al.*^49^ found that Au@Ag/ZIF-8 exhibited a strong bimetallic synergistic effect and high catalytic activity. Chao Hou *et al.*^50^ synthesized Rh@ZIF-8, a highly efficient catalyst in the hydroformylation of alkenes. Peizhou Li *et al.*^51^ prepared highly dispersed Ni NPs immobilized on ZIF-8 (Ni/ZIF-8) and examined catalytic activity of hydrogen generation in the hydrolysis of ammonia borane. It is interesting to note that turnover frequency (TOF) values obtained for Ni/ZIF-8 are among the highest values for Ni catalysts ever reported^52^. However, there is little or no research on transition metals supported on ZIF-8 as a catalyst for hydrocracking of Jatropha oil.

Some literature has obtained encapsulation of phosphotungstic acid (PTA) into the MOF, such as MIL-101^53^, MIL-100(Fe)^54^, HKUST-1 Cu_3_(BTC)_2_^55^ and Fe-EMOF^56^. In our previous work, we found that PTA combined with Ni/Al_2_O_3_, Ni/hydroxyapatite and flower-like NiO can be used as effective bifunctional catalysts for the hydrocracking of plant oil^57^,^58^,^59^. The PTA supplies the metal sites (W oxide species) and also provides acid sites for isomerization. Since nonedible Jatropha oil has many advantages, including high oil production at a low price and high energy density^60^,^61^, in this study, we prepared NiO and PTA supported on ZIF-8 as an efficient catalyst for hydrocracking of Jatropha oil. Results show that these catalysts exhibit high catalytic activity, good selectivity, and can prevent sulfurization and its effects on the environment and human health.

## Methods

### Synthesis of NiO-PTA/ZIF-8 catalyst

**Figure 1:**
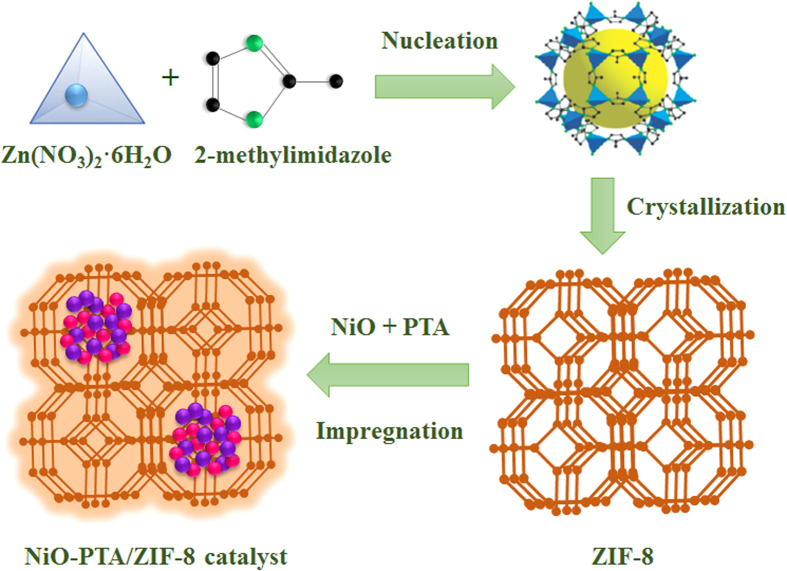
Schematic diagram of synthesis of NiO-PTA/ZIF-8 catalyst (diagram was drawn by Jing Liu).

Figure 1 displays the synthetic procedure of NiO-PTA/ZIF-8 catalyst. Blank ZIF-8 nanocrystals were synthesized according to Christoph Rösler *et al.*^62^. Typically, Zn(NO_3_)_2_·6H_2_O (2.0 g) was added into 40 mL methanol and stirred until it was completely dissolved. Next, 2-methylimidazole (2.0 g) was added into the above solutions and stirred. After 10 min, a white suspension began to form, which was maintained at 25 °C and stirred for a total synthesis time of 2 h. Finally, the mixture was kept at 60 °C for 12 h without stirring. The white precipitates were collected by centrifuging and washed with methanol three times. Nickel nitrate and phosphotungstic acid (PTA) was introduced into the ZIF-8 with an impregnation method. The synthesized ZIF-8 was dried under a vacuum at 150 °C for 5 h and quickly added to a solution of Ni(NO_3_)_2_·6H_2_O (5 wt%). Then the NiO/ZIF-8 was prepared by calcination in a muffle oven at 400 °C for 4 h. Finally, a NiO-PTA/ZIF-8 catalyst was obtained by impregnanting NiO/ZIF-8 with an aqueous solution of H_3_O_40_PW_12_. We set the PTA loading amount 30%, which was confirmed as the suitable PTA loading amount for hydrocracking by our previous work^63^. Impregnated samples were calcined at 200 °C for 3 h.

The NiO-PTA/Al_2_O_3_ catalyst was used to compare with NiO-PTA/ZIF-8 catalyst, which was also prepared by wet impregnation. First, the NiO/Al_2_O_3_ was prepared by impregnating aqueous solutions of Ni(NO_3_)_2_·6H_2_O (5 wt%) on a commercial Al_2_O_3_ (diameter 0.2–0.3 mm). Impregnated samples were dried overnight at 105 °C and calcined at 400 °C for 4 h. The NiO-PTA/Al_2_O_3_ catalyst was prepared by impregnating the Ni/Al_2_O_3_ catalyst with a solution containing 30 wt% PTA. Impregnated samples were calcined at 200 °C for 3 h.

### Characterization of NiO-PTA/ZIF-8 catalyst

Samples were examined under a scanning electron microscope (SEM, Hitachi S-4800) and transmission electron microscope (TEM, JEM-1010). The EDS-mapping (Energy-dispersive X-ray spectroscopy) was performed on a SU8010 instrument (Hitachi High-Tech, Japan). The working parameters of the EDS were: high voltage (HV) 15 kV and work distance (WD) 10 mm, and the sample was sputter coated with gold to reduce charging effects. Nitrogen adsorption-desorption isotherms were carried out a V-Sorb 2800 TP Surface Area and Pore Distribution Analyzer instrument. Before gas analysis, the samples were evacuated for 4 h at 300 °C under a vacuum. The surface area of the samples was estimated using the Brunauer-Emmett-Teller (BET) equation. The total pore volume (V_total_) was determined using the adsorption branch of the N_2_ isotherm at P/P_0_ = 0.99, and the micropore volume (V_micro_) was calculated using the t-plot method. The size distribution was obtained by the Barrett-Joyner-Halenda (BJH) method by analyzing the adsorption branch of the isotherm. Fourier transform infrared (FT-IR) spectra were recorded using KBr pellets on GANGDONG FTIR-650 ranging from 400 to 4000 cm^−1^ at 1.5 cm^−1^ resolution averaging 32 scans. X-ray diffraction (XRD) patterns were recorded on a Bruker D8 Advance X-ray powder diffractometer with Cu Kα radiation (40 mA and 40 kV), over an angular range from 5° to 60°, a scan rate of 2° per minute, and with a step size of 0.02°. X-ray photoelectron spectroscopy (XPS) data was performed on an ESCALab250 electron spectrometer from Thermo Scientific Corporation with monochromatic 150 W Al Kα radiation. The analysis chamber pressure was about 6.5 × 10^−10^ mbar. Analysis of peaks was performed with a weighted sum of Lorentzian and Gaussian component curves after background subtraction. Binding energies were calibrated using the C 1 s photoelectron peak at 284.8 eV as a reference.

### Catalytic properties

The experimental rig for catalyst testing was basically the same as our previous work^64^. The catalyst (1.0 g) was packed into a custom designed stainless steel tubular reactor with an inner diameter of 1.2 cm and a length of 56 cm. Prior to the reaction, the catalyst was pretreated by reduction with 3 MPa of H_2_ at 400 °C for 3 h. Then the reactant Jatropha oil was fed using a high-pressure liquid pump into the reactor. The reaction conditions for the hydrotreatment experiment were as follows: temperature 360 °C, hydrogen pressure 3 MPa, liquid hourly space velocity (LHSV) 9 h^−1^, and H_2_/feed 1000 mL/mL. Liquid products were collected in a liquid-gas separator and analyzed by gas chromatograph^65^,^66^,^67^,^68^,^69^ (GC-900C) equipped with a capillary column (AT. SE-30, 30 m × 0.25 mm; carrier gas N_2_) and a flame ionization detector (FID). The product oil (0.2 μL) was loaded onto the column and the following temperature program was used: initial column temp. 60 °C for 2 min, heating rate 15 °C /min to 210 °C, then at 8 °C /min to 270 °C, and at 5 °C /min to 285 °C, with a dwelling time of 5 min at 285 °C. Individual products were identified by GC standards. The conversion of Jatropha oil was calculated as:


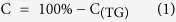


where C_(TG)_ is the concentrations of triglycerides (%) in the product oil, determined by GC analysis. The selectivity of C15-C18 hydrocarbon was calculated as:


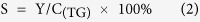


where Y is the yield of the C15-C18 hydrocarbon (%), determined by GC analysis, and C is the conversion of Jatropha oil (%), calculated with Eq. (1).

## Results

### Catalyst characterization

**Figure 2:**
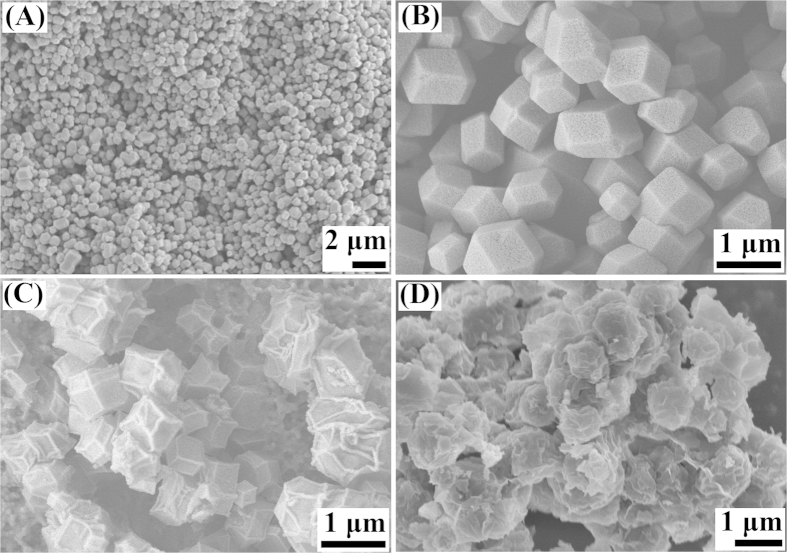
SEM images of ZIF-8 (**A,B**) at different magnifications, NiO-PTA/ZIF-8 catalyst (**C**) and spent NiO-PTA/ZIF-8 catalyst (**D**).

As shown in Fig. 2, SEM images confirm the microscopic architecture of the composites. ZIF-8 was homogeneous, with a hexagonal shape, and the average particle size was about 500 nm. There were no obvious aggregations and changes in size and morphology when the NiO-PTA supported on ZIF-8, reflecting their good stability. However, the hexagonal shape of the spent catalyst showed small change, since carbon deposition and crystals significantly aggregated after the reaction.

Energy dispersive X-ray spectroscopy (EDS) was carried out to examine chemical the composition of the NiO-PTA/ZIF-8 catalyst. Figure S1 in the supplementary information shows the expected elemental constituents of Zn, O, Ni, P and W. EDS mapping also reveals that Ni, P and W elements were homogeneously distributed on the ZIF-8 substrate. The estimated Ni, P and W amount was 0.930 atom%, 1.108 atom% and 2.701 atom%, respectively. Compared with fresh catalyst, the Ni, P and W content of spent catalyst was 0.734 atom%, 0.971 atom% and 2.623 atom%, respectively, which decreased slightly after reaction.

To further investigate the morphologies of the ZIF-8 and NiO-PTA/ZIF-8 catalyst, TEM analysis was performed. As shown in Fig. S2A,B (see supplementary information), the ZIF-8 and NiO-PTA/ZIF-8 catalyst both showed the typical hexagonal shape with a particle size around 500 nm, in accordance with that observed in SEM images. This indicates that impregnation of NiO-PTA did not result in an obvious change in particle morphology for ZIF-8 particles. TEM images of the NiO-PTA/ZIF-8 catalyst (Fig. S2C) clearly showed surface clumping of NiO and PTA nanoparticles which form large agglomerated particles. The existing synergistic effects between the NiO-PTA and ZIF-8 support are discussed in the XPS results below.

**Figure 3:**
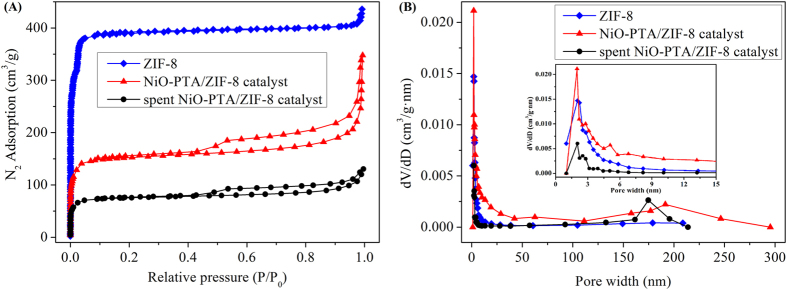
N_2_ adsorption-desorption isotherms (**A**) and pore size distributions based on the BJH method (**B**) inset: enlarge Figure) of ZIF-8, NiO-PTA/ZIF-8 catalyst and spent NiO-PTA/ZIF-8 catalyst.

The surface area and pore distribution of ZIF-8, NiO-PTA/ZIF-8 catalyst and spent NiO-PTA/ZIF-8 catalyst were analyzed using nitrogen adsorption-desorption isotherms (Fig. 3). The isotherms of the three samples (Fig. 3A) showed an abrupt increase at low relative pressure (P/P_0_ < 0.1), indicating their microporous structure. The pure ZIF-8 displayed typical type-I behavior^40^. The NiO-PTA/ZIF-8 catalyst and spent NiO-PTA/ZIF-8 catalyst both displayed typical type-IV isotherms with a type H_4_ hysteresis loop in the range of P/P_0_ = 0.4–0.8, indicating the presence of mesopores^70^. It also displayed high adsorption capacities at high relative pressure (P/P_0_ > 0.8), demonstrating the coexistence of macropores and mesopores^71^. These findings indicate that the NiO-PTA/ZIF-8 catalyst possesses hierarchically micro-meso-macroporous textures. This may be due to damage to the pores caused by thermal decomposition of the Ni precursor^72^. Table 1 displays the BET surface area, total pore volume and micropore volume of synthesized ZIF-8 nanoparticles at 1724.19 m^2^/g, 0.67 cm^3^/g and 0.58 cm^3^/g, respectively. Compared with ZIF-8, the NiO-PTA/ZIF-8 catalyst exhibited decreased BET surface area of 611.21 m^2^/g and a micropore volume of 0.21 cm^3^/g due to blocking of cavity windows by the deposited NiO and PTA on the surface^73^. After the reaction, BET surface area and total pore volume of spent NiO-PTA/ZIF-8 catalyst had both decreased. This was likely due to agglomeration of abundant deposited coke, leading to blockage of some micropores and mesopores.

The pore size distributions calculated by the BJH method are shown in Fig. 3B. The pore sizes were narrowly distributed between 1 nm and 5 nm, indicating that ZIF-8 nanoparticles had a highly uniform pore structure. However, the pore size distributions widened, and larger pore diameters distinctly increased after NiO and PTA were added. Macropores formed in the range of 150–300 nm for the NiO-PTA/ZIF-8 catalyst, implying that deposition of NiO-PTA and corresponding calcination might affect not only the surface area, but also pore diameter. The macropores can also be seen in spent NiO-PTA/ZIF-8 catalyst, which were not blocked by deposited carbon.

**Figure 4:**
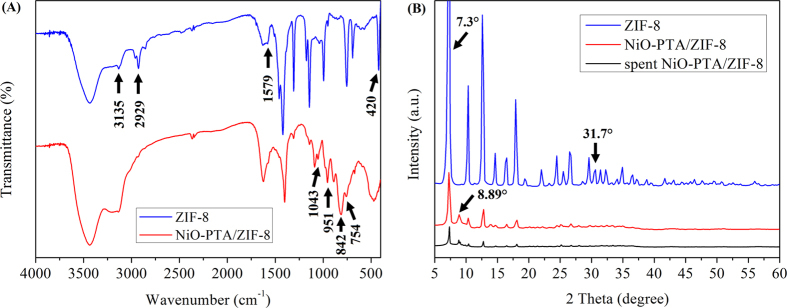
FTIR spectra (**A**) and XRD patterns (**B**) of ZIF-8, NiO-PTA/ZIF-8 catalyst and spent catalyst.

Figure 4A present the FT-IR spectrum of ZIF-8 and NiO-PTA/ZIF-8 catalyst with an absorption region of 400–4000 cm^−1^. Several bands were observed for ZIF-8 in the FT-IR spectrum. For example, absorption bands at 2929 cm^−1^ and 3135 cm^−1^ were attributed to the aromatic and aliphatic C-H stretch of the imidazole, respectively^74^. The band at 1579 cm^−1^ could be assigned as the C = N stretch mode^75^. The absorption band at 420 cm^−1^ was observed for the Zn-N stretching mode, while those in the 1100–1400 cm^−1^ region were associated with the C-N stretch^76^. Compared to the pure ZIF-8, the FT-IR spectra of the NiO-PTA/ZIF-8 catalyst showed the absorption bands at 1043 cm^−1^, 951 cm^−1^, 842–875 cm^−1^ and 754 cm^−1^, respectively. This corresponds to the *ν*(P-Oa), terminal *ν*(W-Ot), corner-sharing *ν*(W-Ob) and edge-sharing *ν*(W-Oc) band vibrations of H_3_PW_12_O_40_^77^,^78^. The band at 403 cm^−1^ was assigned to the Ni-O stretching mode, which was difficult to observe. Bands below 600 cm^−1^ were due to the symmetric stretching vibration of metal-oxygen groups^79^, while ZIF-8 showed no apparent absorption in this region. These facts suggest that NiO and PTA were present in the catalysts. The presence of H_2_O in both samples was likely due to moisture adsorption when exposed to air.

Figure 4B showed XRD patterns of the ZIF-8, NiO-PTA/ZIF-8 and spent catalyst. Almost all of the XRD peaks for ZIF-8 were in good agreement with previous reports^80^, confirming the formation of pure crystalline ZIF-8 phase. A minimal amount of ZnO in the impurity phase was found at 31.7° 81. As expected, all three samples showed the characteristic reflection at 2θ = 7.3°, attributed to the 110 peak of ZIF-8 82. The powder XRD pattern showed no significant change in the crystalline structure of both NiO-PTA/ZIF-8 and spent catalyst, with respect to the plain ZIF-8. This indicates that the framework of ZIF-8 was stable throughout the entire process of catalyst preparation and catalytic reaction. However, the large amount of NiO and PTA dispersed at the molecular level in ZIF-8 led to a marked decrease in intensity at 7.3°, and the spent NiO-PTA/ZIF-8 catalyst showed more decrease at 7.3° due to carbon deposition. The XRD pattern of NiO-PTA/ZIF-8 catalyst did not show the characteristic diffraction peaks of NiO, suggesting that the NiO were highly dispersed on ZIF-8 83. An alternative explanation was that the signal was below the detection limit due to relatively low NiO loading^50^. In Fig. 3B the positions of the sharp peaks below 2θ = 10° (8.89°) could be ascribed to cubic phase H_3_PW_12_O_40_, and were indexed to the reported data (JCPDS 50-0304)^84^.

**Figure 5:**
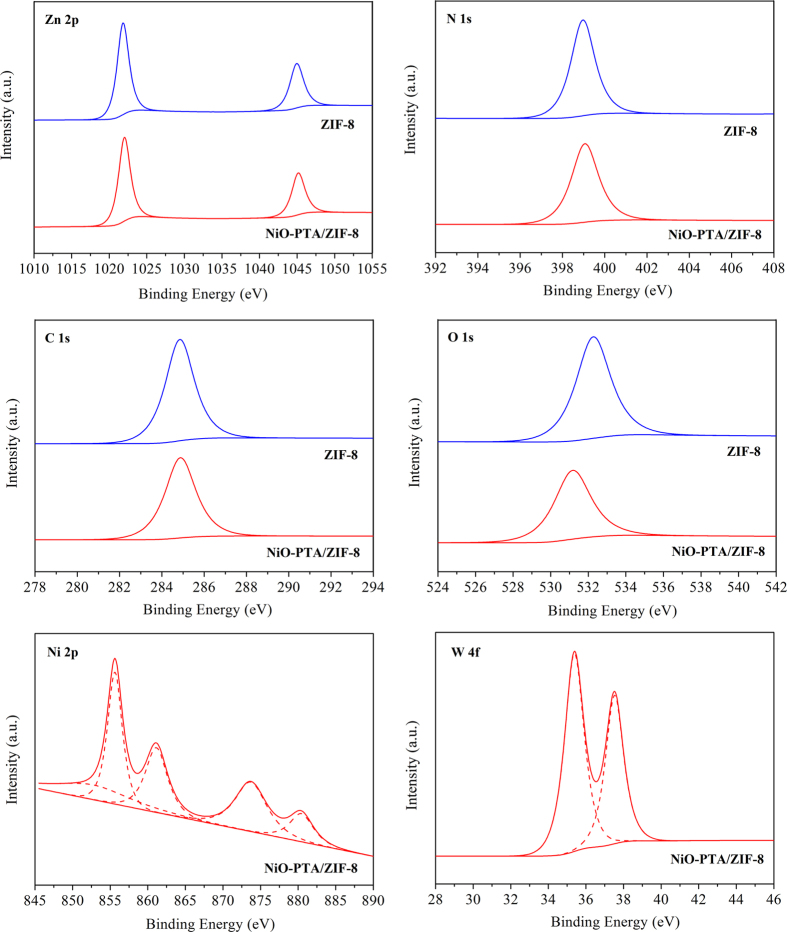
XPS spectrum of ZIF-8 and NiO-PTA/ZIF-8 catalyst.

Figure 5 shows elemental surveys by X-ray photoelectron spectroscopy (XPS), which showed the presence of the ZIF-8 framework elements (Zn, C, N) and O in ZIF-8 sample and clearly confirmed the presence of not only Zn, C, N and O but also Ni and W in the NiO-PTA/ZIF-8 catalyst sample. The binding energy of 1021.82 eV corresponded to the Zn 2p_3/2_, while the other located at 1044.93 eV was attributed to the Zn 2p_1/2_^85^. The binding energy distance between these two lines was 23.11 eV, indicating that the Zn ions in the composites were of +2 states^51^. After loading with NiO and PTA, the peak for Zn 2p shifted slightly to higher binding energy (from 1021.82 to 1022.02 and 1044.93 to 1045.16 eV, respectively), implying that the Zn were involved in NiO and PTA sorption^86^, and the weak binding or coordination of Zn in ZIF-8 to NiO and PTA^87^. As shown in Fig. 5, both samples exhibited a symmetric peak (about 399.06 eV) for the nitrogen on imidazole of the ZIF-8 nanoparticles^88^, indicating that there was only one form of nitrogen. The C 1 s peaks of two samples for the N-doped carbon materials were observed at about 284.85 eV, consistent with sp^2^ carbons^89^. In the O 1 s XPS spectrum, the bonding energy of ZIF-8 was peaked at 532.28 eV, which could be assigned to O^2−^ ions^90^ in the Zn-O bonding of the wurtzite ZnO structure, which has been also observed in XRD results. After adding NiO and PTA, the bonding energy was decreased from 532.28 to 531.17 eV. Such a negative shift could be due to the rich electron density on the oxygen atoms arising from the Zn-O linkage in the catalyst. In Fig. 5, two peaks for the 2p_3/2_ and 2p_1/2_ components at 855.62 eV and 873.70 eV were characteristics of the NiO phase and were in accordance with the literature^91^. Furthermore, the presence of two shakeup satellite peaks (861.14 eV and 880.44 eV for satellite of Ni 2p_3/2_ and Ni 2p_1/2_, respectively) indicated the electronic state of the Ni^2+^ ion^92^. This observation confirmed that the NiO-PTA/ZIF-8 catalyst contained more oxo-Ni species due to the larger peak areas. Notably, the peak at 852.73 eV was ascribed to metallic Ni^93^. This could be due to the reduction of nickel ions, while the positive shifts in binding energies of the Zn 2p states were ascribed to the oxidation of zinc ions. This implies that Zn species might donate partial electrons to Ni oxide species, the electron transfer between Zn and Ni due to the NiO-loaded and calcination. As shown in Fig. 5, the W 4f spectrum contained a doublet at a binding energy of 35.38 and 37.52 eV, assigned to W 4f_7/2_ and W 4f_5/2_ lines, respectively, which were characteristic of the W^6+^ ion^94^. This result clearly showed the existence of assembled NiO and PTA on NiO-PTA/ZIF-8 catalyst. Furthermore, the surface atomic contents of the NiO-PTA/ZIF-8 catalyst were 0.84% for Ni and 2.53% for W. This indicates that the catalyst was comprised of more tungsten atoms and was deficient in nickel atoms on the surface, which is in line with the EDS measurement.

### Catalytic properties of NiO-PTA/ZIF-8 catalyst in hydrocracking of Jatropha oil

**Figure 6:**
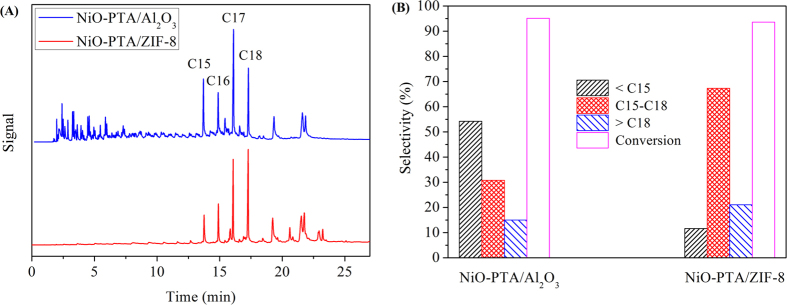
GC of products obtained from hydrocracking of Jatropha oil (**A**) and comparison to the selectivity of products and conversion of Jatropha oil (**B**) over the NiO-PTA/Al_2_O_3_ and NiO-PTA/ZIF-8 catalyst (360 °C and 30 wt%PTA).

Figure 6A displays GC chromatograms of product oil from NiO-PTA/Al_2_O_3_ catalyst and NiO-PTA/ZIF-8 catalyst. Results show that the major components in both product oils were C15H32, C16H34, C17H36, and C18H38. Heptadecane and octadecane were the two most abundant liquid alkanes, which were produced from the fatty acids of the triglycerides. Although the product of C17H36 and C18H38 over NiO-PTA/Al_2_O_3_ catalyst produced more *Iso*-alkanes, this catalyst also had more light fraction (<C15) than that over NiO-PTA/ZIF-8 catalyst. These reactions are undesirable in the hydroconversion of triglycerides, because the by-products formed block the reaction sites and also act as sites for coke formation on the surface of the catalyst^3^. They both showed heavy fraction (>C18), which mainly contained long chain esters formed by esterification of alcohol and free fatty acid^95^, or stable oxygenated intermediate products such as esters and some acids, alcohols, aldehydes and ethers^96^.

Figure 6B displays the selectivity of product oil and conversion of Jatropha oil over the two catalysts. Although the Jatropha oil conversions over NiO-PTA/Al_2_O_3_ catalyst was close to the NiO-PTA/ZIF-8 catalyst (both more than 90%), the C15-C18 hydrocarbon selectivity over the NiO-PTA/ZIF-8 catalyst (67.31%) was much more than that of the NiO-PTA/Al_2_O_3_ catalyst (30.78%), and the selectivity increased 36.53% by NiO-PTA/ZIF-8 catalyst. We speculated that hydrocarbons less than C15 were probably adsorbed and detained in the micropore, and could not diffuse from the catalyst. This resulted in an increase of C15-C18 hydrocarbon selectivity. Moreover, appropriate amounts of macropores would favor the impregnation of large-size PTA molecules and readily allow the diffusion of bulky triglyceride molecules and prevent pore blockage.

The stability of the NiO-PTA/ZIF-8 catalyst was investigated in a continuous flow reactor at 360 °C, 3 MPa, LHSV 9 h^−1^ (Table 2). The selectivity of products and conversion of Jatropha oil measured at 15, 44, 76 and 104 h on stream, and the catalyst was stable over the entire duration. Results show that Jatropha oil conversion declined by 17%; however, this was still more than 75% during the 104 h. The liquid hydrocarbon (C15-C18) selectivity remained constant. This indicates that there was no significant deactivation of the catalyst with reaction time.

### Reaction kinetics

**Figure 7:**
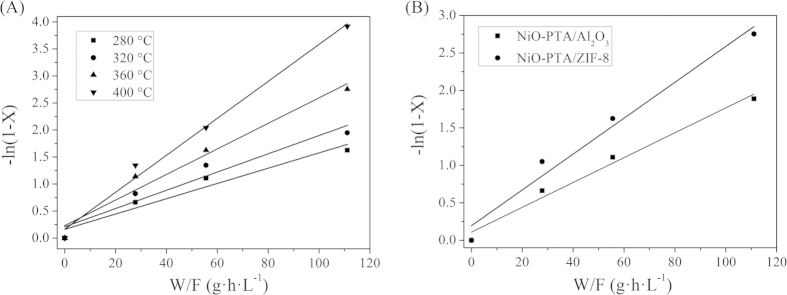
Plot of –ln(1 − X) vs W/F for hydrocracking of Jatropha oil under 280–400 °C over NiO-PTA/ZIF-8 catalyst (**A**) and pseudo-first-order plots of NiO-PTA/Al and NiO-PTA/ZIF-8 catalyst under 360 °C, 3.0 Mpa (**B**).

A Langmuir-Hinshelwood model was used in the first kinetic study on the hydrocracking of Jatropha oil over different catalysts below 360 °C, 3.0 MPa and 9–36 h^−1^. The following first-order equation was obtained^97^:


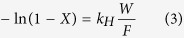


where X is the conversion of triglyceride to the hydrocarbon products, k_H_ (h^−1^) is the hydrocracking rate constant, W (g) is the weight of the catalyst and F (L/h) is the flow rate of triglyceride. The essentially linear relationship of −ln(1 − X) vs. reciprocal space velocity and the apparent first-order plot for the hydrocracking of Jatropha oil is shown in Fig. 7. This indicates that the experimental data followed empiric pseudo-first-order-kinetics.

The activity of catalysts in hydrocracking can be described by examining the conversion of triglycerides. This involves evaluating the rate of disappearance of triglycerides, using pseudo-first order kinetics to fit the relevant experimental data for different catalysts at 360 °C and 3.0 MPa^98^. Results are presented in Table 3. The relative pseudo-first order rate constants (k_H_) of NiO-PTA/Al_2_O_3_ and NiO-PTA/ZIF-8 catalyst were calculated, and results show that the k_H_ of NiO-PTA/ZIF-8 catalyst was 1.4-fold higher than that of the NiO-PTA/Al_2_O_3_ catalyst under the same reaction condition. This indicates that the conversion of triglyceride over the NiO-PTA/ZIF-8 catalyst was higher than that of the NiO-PTA/Al_2_O_3_ catalyst. The values of activation energy (E_H_) calculated from the Arrhenius equation [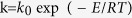]^99^ were summarized in Table 3. Thermodynamic parameters for two catalysts were evaluated for the temperature range of 280–400 °C, while the pressure and LHSV were maintained at 3.0 MPa and 9 h^−1^. E_H_ suggests the relative difficulty of the conversion of triglycerides. As shown in Table 3, the activation energy of conversion of triglycerides over NiO-PTA/ZIF-8 was lower than NiO-PTA/Al_2_O_3_. This indicates that conversion of triglyceride was easier over NiO-PTA/ZIF-8, due to its higher hydrocracking activity. This may be related to the metal active center (Ni and W phase). The NiO-PTA/ZIF-8 catalyst showed a high surface area, high metal content and uniform metal dispersion (proven by BET, XPS and SEM-EDS results), leading to the formation of a more active center and then a marked increase in hydrogenation activity.

Table S1 in Supplementary Information showed the effect of LHSV on the conversions of Jatropha oil and selectivity of products oil over NiO-PTA/ZIF-8 catalyst at reaction temperature 280–400 °C. It can be seen that the conversion and selectivity of C15-C18 both decreases as the LHSV increases from 4.5 to 36 h^−1^ under temperature of 280–360 °C. This decrease is due to the fact that with increasing LHSV, the contact time of the oil on the catalyst decreases, resulting in lower conversion as well as selectivity of main product. However, selectivity of C15-C18 increases when the LHSV increasing, it probably because that cracking ability of alkane increases at 400 °C, it can be observed that the light fractions (<C15) are more than that at 280–360 °C. While, the cracking ability decreases as the LHSV increasing which leads to the selectivity of C15-C18 increase, but the conversions of Jatropha oil are also decreased.

## Discussion

In this study, 10 g NiO-PTA/Al_2_O_3_ catalyst was required to reach the same conversion of Jatropha oil as the NiO-PTA/ZIF-8 catalyst (1.0 g). Compared with the NiO-PTA/Al_2_O_3_ catalyst, the dosage of catalyst decreased 10 times and catalytic efficiency increased 10 times over the NiO-PTA/ZIF-8 catalyst. As shown in Fig. S3 (see supplementary information), the NiO-PTA/Al_2_O_3_ also exhibited Langmuir type IV isotherms with a typical mesoporous material and size-homogeneous 1D slit channels^100^,^101^. The total pore volume and micropore volume of NiO-PTA/Al_2_O_3_ were 0.39 cm^3^/g and 0.08 cm^3^/g, which were both smaller than NiO-PTA/ZIF-8 catalyst (0.54 cm^3^/g and 0.21 cm^3^/g). The BET surface area of NiO-PTA/Al_2_O_3_ was only 223 m^2^/g, while that of the NiO-PTA/ZIF-8 catalyst was more than 600 m^2^/g. Generally, the catalysts at a nanosize scale with a high surface area should provide the catalysts with higher activity^102^. Thus, the NiO-PTA/ZIF-8 catalyst possessed more active sites for hydrocracking of Jatropha oil than the NiO-PTA/Al_2_O_3_ catalyst. As confirmed by XPS results, the surface atomic contents of the NiO-PTA/ZIF-8 catalyst were 0.84% for Ni and 2.53% for W, and 0.41% for Ni and 1.29% for W of NiO-PTA/Al_2_O_3_ catalyst. We also prepared another sample of NiO-PTA/ZIF-8 catalyst with similar atomic content (0.53% for Ni and 1.36% for W) to NiO-PTA/Al_2_O_3_ catalyst, as shown in Fig. S3. The BET surface area, total pore volume and micropore volume of the NiO-PTA/ZIF-8 catalyst were 814 m^2^/g, 0.47 cm^3^/g and 0.28 cm^3^/g, respectively. These figures were still larger than that of the NiO-PTA/Al_2_O_3_ catalyst. GC results are showed in Fig. S4 (see supplementary information). The Jatropha oil conversions over the NiO-PTA/ZIF-8 catalyst was 92.55%, which was close to that of the NiO-PTA/Al_2_O_3_ catalyst. However, the C15-C18 hydrocarbon selectivity of the NiO-PTA/ZIF-8 catalyst (53.76%) was also more than that of the NiO-PTA/Al_2_O_3_ catalyst (30.78%). Therefore, pore structure as well as the surface area played an important role in the activity of the catalyst.

In conclusion, a highly efficient NiO-PTA/ZIF-8 catalyst was successfully prepared through the impregnation method. Results show the prominently enhanced selectivity of C15-C18 hydrocarbon (67.31%) for hydrocracking of Jatropha oil compare to the NiO-PTA/Al_2_O_3_ catalyst. Product selectivity increased 36.53% due to ZIF micropore structure, and the catalytic efficiency increased 10 times with the NiO-PTA/ZIF-8 catalyst due to its high catalyst surface area. The NiO-PTA/ZIF-8 catalyst exhibited excellent performance and stability in the reaction. The kinetic behavior of two catalysts was examined with an assumption of pseudo-first-order reaction kinetics. This work sheds light on new opportunities in the development of other metal particles (e.g., Pt and W), which have wide potential applications in catalytic reactions. Since non-sulfided NiO-PTA/ZIF-8 catalyst can bypass the presulfurization process, shows promise as an alternative to sulfided catalysts for green diesel production.

## Additional Information

**How to cite this article**: Liu, J. *et al.* NiO-PTA supported on ZIF-8 as a highly effective catalyst for hydrocracking of Jatropha oil. *Sci. Rep.* **6**, 23667; doi: 10.1038/srep23667 (2016).

## Supplementary Material

Supplementary Information

## Figures and Tables

**Table 1:** Textural properties of ZIF-8 and catalysts^a^.

Samples	S_BET_ (m^2^/g)	V_total_ (cm^3^/g)	V_micro_ (cm^3^/g)
ZIF-8	1724.19	0.67	0.58
NiO-PTA/ZIF-8 catalyst	611.21	0.54	0.21
spent NiO-PTA/ZIF-8 catalyst	305.63	0.20	0.11

^a^S_BET_, BET surface area; V_total_, total pore volume; V_micro_ micropore volume.

**Table 2:** Stability test of NiO-PTA/ZIF-8 catalyst.

Run time (h)	15	44	76	104
Conversion (%)	80.58	81.29	76.43	76.25
Selectivity (%)	65.32	65.77	61.60	62.47

**Table 3:** Kinetic and thermodynamic parameters for triglyceride-conversion of hydrocracking of Jatropha oil.

Catalysts	k_H_ (h^−1^)^a^	E_H_ (kJ/mol)^b^
NiO-PTA/Al_2_O_3_	0.01657	22.24
NiO-PTA/ZIF-8	0.02392	15.26

^a^Reaction temperature 360 °C, H_2_ pressure 3.0 MPa, 9–36 h^−1^.

^b^Reaction temperature 280–400 °C, H_2_ pressure 3.0 MPa, 9 h^−1^.
