# Supplementary material for: NiO-PTA supported on ZIF-8 as a highly effective catalyst for hydrocracking of Jatropha oil
Source: Sci Rep. 2016 Mar 29;6:23667. doi: 10.1038/srep23667 (PMC4810320; doi:10.1038/srep23667)
Supplement: Supplementary Information [file srep23667-s1.pdf]

Supplementary Information

NiO-PTA supported on ZIF-8 as a highly effective catalyst for hydrocracking of Jatropha oil

Jing Liu [1,*], Jing He [1], Luying Wang [1], Rong Li [1], Pan Chen [1], Xin Rao [1], Lihong Deng [1], Long Rong [2], Jiandu Lei [1,*]

[1] *Beijing Key Laboratory of Lignocellulosic Chemistry*, College of Materials Science and Technology, Beijing Forestry University, Beijing 100083, P. R. China

[2] Key Laboratory for Biomechanics and Mechanobiology of Ministry of Education, School of Biological Science and Medical Engineering, Beihang University, Beijing 100191, P. R. China

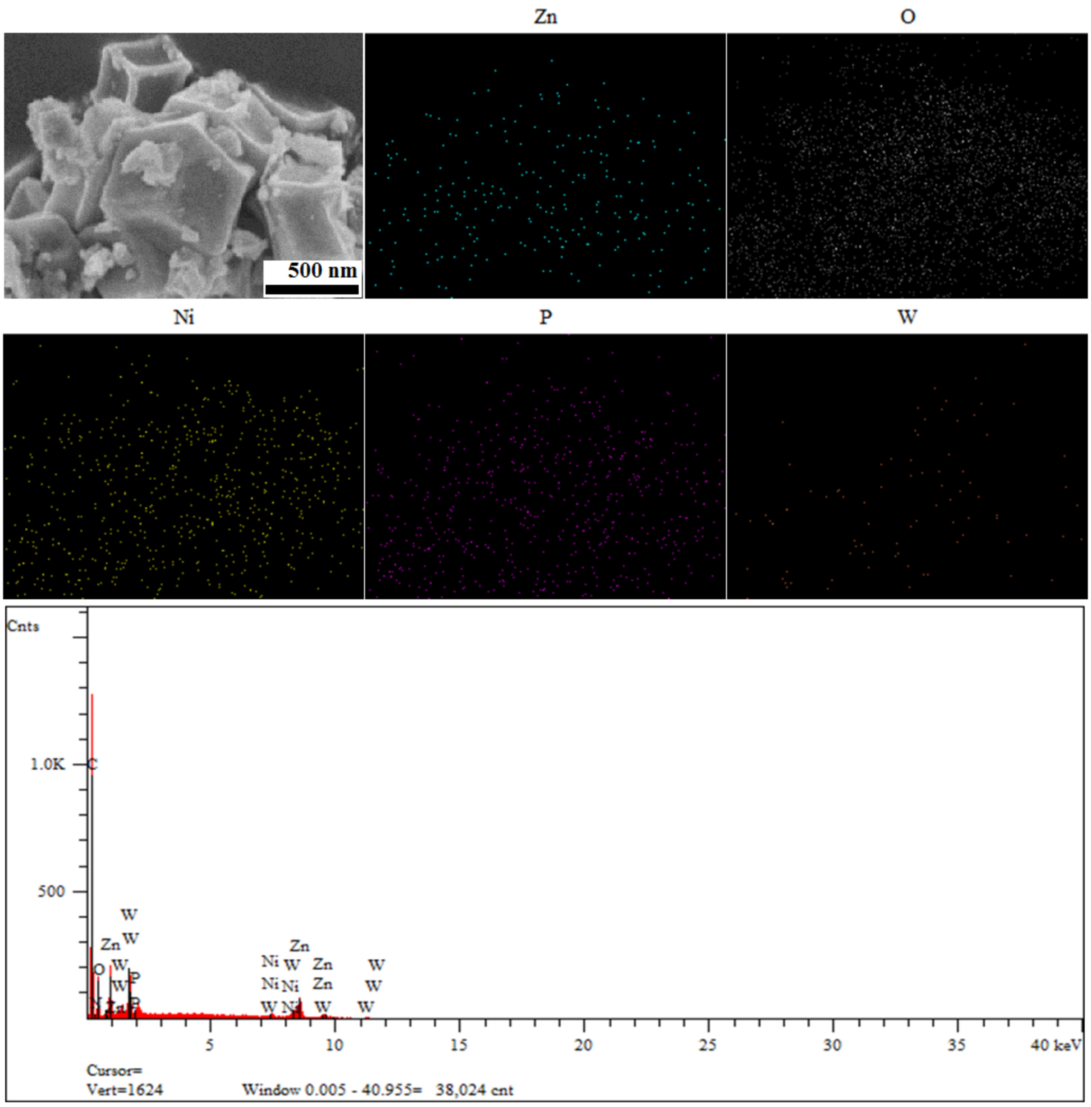

Figure S1 SEM-EDS surface mapping of Zn, O, Ni, P and W element at NiO-PTA/ZIF-8 catalyst.

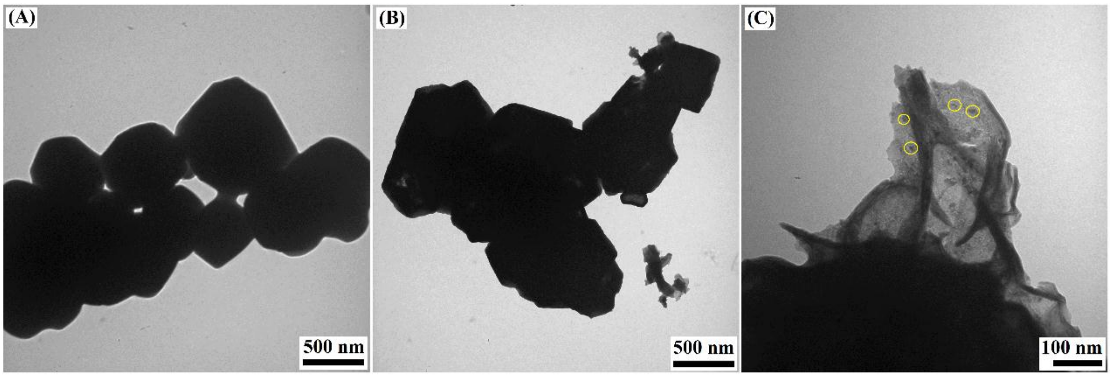

Figure S2 TEM image of ZIF-8 (A) and NiO-PTA/ZIF-8 catalyst (B and C).

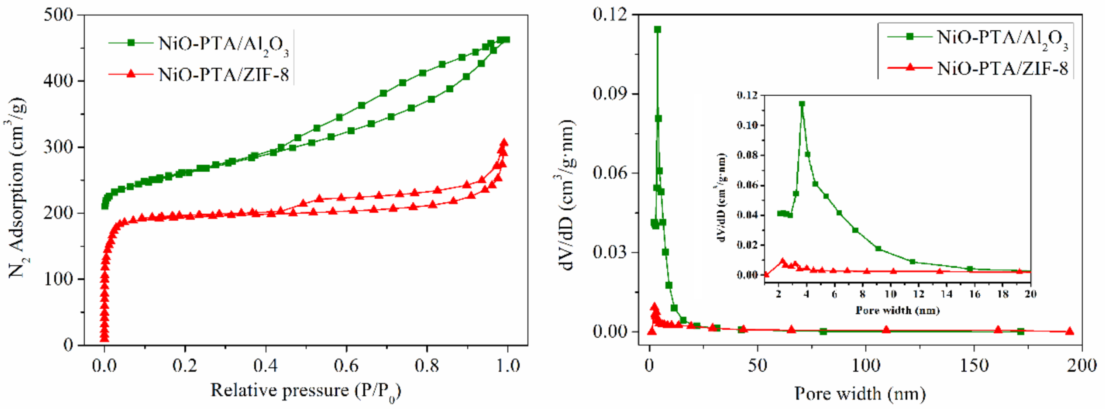

Figure S3 N_2 adsorption-desorption isotherms (A) and pore size distributions based on BJH method (B, inset: enlarge Figure) of NiO-PTA/Al_2O_3 and NiO-PTA/ZIF-8 with similar atomic content.

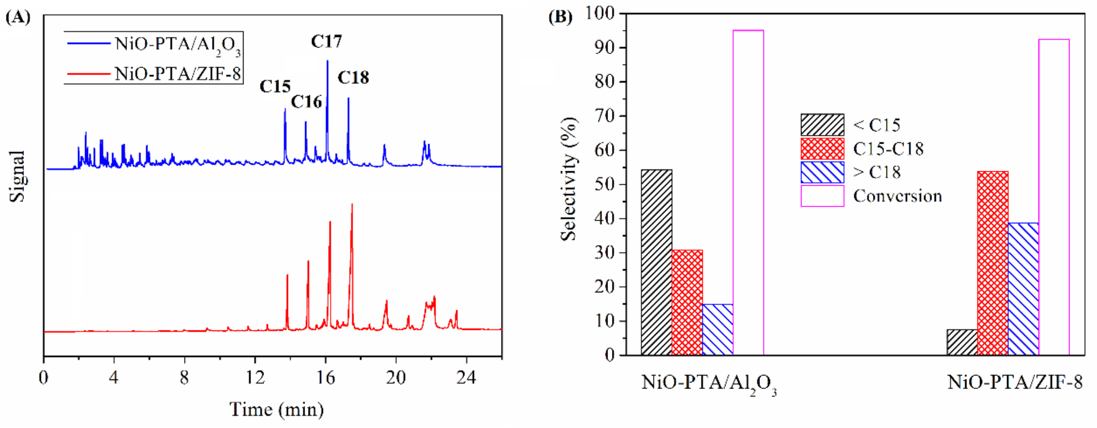

Figure S4 GC of the products obtained from hydrocracking of Jatropha oil (A) and comparison to the selectivity of products and conversion of Jatropha oil (B) over NiO-PTA/Al_2O_3 and NiO-PTA/ZIF-8 catalyst with similar atomic content (360 ℃).

Table S1 Effect of LHSV on the conversions of Jatropha oil and selectivity of products oil over NiO-PTA/ZIF-8 catalyst at reaction temperature 280-400 ℃, H_2 pressure 3.0 MPa

Temperature (℃)	LHSV (h^{-1})	Conversion (%)	Selectivity (%)		
			< C15	C15-C18	> C18
280	4.5	85.28	1.16	61.99	36.85
	9	80.31	1.45	60.68	37.87
	18	67.25	1.39	54.07	44.54
	36	48.4	1.25	39.12	59.63
320	4.5	90.62	5.83	67.45	26.72
	9	85.74	6.67	65.3	28.03
	18	74.83	7.12	53.34	39.54
	36	56.02	5.96	37.15	56.89
360	4.5	96.55	7.59	70.18	22.23
	9	93.63	11.64	67.31	21.05
	18	80.3	14.22	55.67	30.11
	36	65.21	12.6	39.86	47.54
400	4.5	99.79	37.54	51.77	10.69
	9	98.01	32.68	55.07	12.25
	18	87.53	24.9	58.48	16.62
	36	74.68	16.82	63.72	19.46
